# Relation between tumor treating fields usage rate ≥75% for the first 3 months and progression-free survival in patients with newly diagnosed WHO grade 4 gliomas

**DOI:** 10.1093/noajnl/vdaf203

**Published:** 2025-09-23

**Authors:** Shoichi Deguchi, Masashi Kinoshita, Fumiharu Ohka, Koichi Mitsuya, Shigeo Ohba, Noriyuki Nakayama, Shinichiro Koizumi, Yu Fujii, Takahiro Yamauchi, Yotaro Kitano, Hiroshi Yamada, Takahiro Nakura, Takahiro Tomita, Yuichi Hirose, Tsuyoshi Izumo, Kazuhiko Kurozumi, Tetsuyoshi Horiuchi, Ken-Ichiro Kikuta, Hidenori Suzuki, Mitsuhito Mase, Shigeru Miyachi, Satoshi Kuroda, Mitsutoshi Nakada, Ryuta Saito

**Affiliations:** Department of Neurosurgery, Nagoya University Graduate School of Medicine, Aichi, Japan; Department of Neurosurgery, Division of Neuroscience, Graduate School of Medical Science, Kanazawa University, Ishikawa, Japan; Department of Neurosurgery, Nagoya University Graduate School of Medicine, Aichi, Japan; Division of Neurosurgery, Shizuoka Cancer Center, Shizuoka, Japan; Department of Neurosurgery, Fujita Health University School of Medicine, Aichi, Japan; Department of Neurosurgery, Gifu University Graduate School of Medicine, Gifu, Japan; Department of Neurosurgery, Hamamatsu University School of Medicine, Shizuoka, Japan; Department of Neurosurgery, Shinshu University School of Medicine, Nagano, Japan; Department of Neurosurgery, University of Fukui, Fukui, Japan; Department of Neurosurgery, Mie University Graduate School of Medicine, Mie, Japan; Department of Neurosurgery, Nagoya City University Graduate School of Medical Sciences, Aichi, Japan (H.Y., M.M.); Department of Neurological Surgery, Aichi Medical University, Aichi, Japan (T.N., S.M.); Department of neurosurgery, University of Toyama, Toyama, Japan (T.T., S.K.); Department of Neurosurgery, Fujita Health University School of Medicine, Aichi, Japan; Department of Neurosurgery, Gifu University Graduate School of Medicine, Gifu, Japan; Department of Neurosurgery, Hamamatsu University School of Medicine, Shizuoka, Japan; Department of Neurosurgery, Shinshu University School of Medicine, Nagano, Japan; Department of Neurosurgery, University of Fukui, Fukui, Japan; Department of Neurosurgery, Mie University Graduate School of Medicine, Mie, Japan; Department of Neurosurgery, Nagoya City University Graduate School of Medical Sciences, Aichi, Japan (H.Y., M.M.); Department of Neurological Surgery, Aichi Medical University, Aichi, Japan (T.N., S.M.); Department of neurosurgery, University of Toyama, Toyama, Japan (T.T., S.K.); Department of Neurosurgery, Division of Neuroscience, Graduate School of Medical Science, Kanazawa University, Ishikawa, Japan; Department of Neurosurgery, Nagoya University Graduate School of Medicine, Aichi, Japan

**Keywords:** multicenter retrospective study, progression-free survival, tumor treating fields

## Abstract

**Background:**

Tumor treating fields (TTFields) therapy is an important treatment for glioblastoma. However, there have been few reports showing the effectiveness thereof in clinical settings worldwide. The aim of this study was to examine the efficacy of TTFields therapy by comparing patients with compliance rates above 75% with those below 75% during the first 3 months of treatment.

**Methods:**

Data were retrospectively collected from electronic medical records for consecutive patients with newly diagnosed World Health Organization grade 4 gliomas who received TTFields therapy at 12 institutes in Japan from January 2018 to December 2023.

**Results:**

In total, 157 patients received TTFields therapy. We analyzed 116 patients who used TTFields for at least the first 3 months. The median age was 57 years; 67 patients were male; and the median KPS before the start of adjuvant temozolomide was 90. The average compliance rate was 78.3%. The median progression-free survival (PFS) and overall survival (OS) were 11.9 months and 23.7 months, respectively. A high compliance rate of TTFields ≥75% for the first 3 months was achieved in 67 patients and was a significant prognostic factor on PFS (*P* = .04). The median PFS for patients with high and low compliance rates was 12.6 and 9.0 months, respectively; the median OS was 25.3 and 21.3 months, respectively (*P* = .15).

**Conclusions:**

TTFields use rate ≥75% in the first 3 months was significantly associated with improved PFS. There was a tendency for OS to be extended. Further analysis with a larger number of cases is required.

Key Points

- We analyzed 116 patients who used TTFields at 12 institutes for at least 3 months.
- High TTFields usage rate was associated with improved progression-free survival.
- High TTFields usage rate also tended to extend overall survival.

Importance of the Study

Tumor treating fields (TTFields) therapy has been an important treatment option for glioblastoma. However, there have been few reports showing its effectiveness in clinical settings worldwide. We performed a retrospective analysis of 116 patients treated by TTFields for at least 3 months across 12 institutes. Multivariate analysis of progression-free survival showed that TTFields usage rate ≥75% in the first 3 months was a significantly favorable factor. In addition, there was a tendency for overall survival to be extended. Our findings may provide patients undergoing TTFields therapy with specific targets regarding treatment duration and daily wear time.

Tumor treating fields (TTFields) therapy is an important treatment option for glioblastoma and inhibits tumor cell proliferation by disrupting mitotic spindle formation during metaphase with low-intensity, medium-frequency, 150-200 kHz alternating electric fields.^1^^,^^2^ The phase 3 EF-14 clinical study demonstrated that adding TTFields therapy to maintenance temozolomide (TMZ) therapy for newly diagnosed glioblastoma (ndGB) significantly extended progression-free survival (PFS) and overall survival (OS).^3^ In addition, there were no serious side effects, although skin disorders were observed.^4^ Based on these results, the US Food and Drug Administration approved TTFields therapy in 2015 as an adjuvant treatment for ndGB patients after completion of surgery and chemoradiation. TTFields therapy was also approved in Japan in December 2017. However, adoption thereof has been limited in real-world settings, and there are few reports analyzing the efficacy of TTFields therapy in actual clinical practice worldwide.

A single-center retrospective study in the United states comparing 37 ndGB patients treated with TTFields and 67 without TTFields did not observe a prolongation of OS.^5^ A single-center retrospective study in China comparing 13 patients with ndGB treated with TTFields and 39 without TTFields showed a significant prolongation of PFS but not of OS.^6^ Recently, a retrospective multicenter study in Japan comparing 165 patients with ndGB treated with TTFields and 165 without TTFields showed no substantial impact on PFS or OS using propensity score-matched analysis.^7^ All these reports compared patients who used TTFields and those who did not, and selection bias was noted as a limitation.

There have also been few reports that retrospectively analyzed the effects of TTFields specifically for patients who used TTFields at a high compliance rate over a certain period. We, therefore, aimed to examine the efficacy of TTFields therapy by comparing patients with compliance rates above 75% with those below 75% during the first 3 months of treatment.

## Materials and Methods

### Ethics

All analyses were approved by the Institutional Review Board of Nagoya University (approval number: 2024-0164), and the study was conducted in accordance with the principles of the Declaration of Helsinki. The need for written informed consent was waived by the institutional review board of Nagoya University owing to the retrospective nature of the study. Participants were given information about the conduct of the study, including its purpose, on a website, and were given the option to opt out of the study whenever possible.

### Patient Population

This retrospective cohort study was conducted at 11 academic centers and a cancer center in the Chubu region of Japan. We retrospectively collected and analyzed the data from 157 consecutive patients who underwent TTFields therapy between June 2018 and December 2023. We included patients aged 20 years or older who had histologically confirmed and newly diagnosed with grade 4 gliomas by the World Health Organization (WHO) Classification of Tumors of the Central Nervous System, fifth edition. We included the patients who underwent radiation therapy with concomitant TMZ. Patients who received carmustine wafers were also included. We excluded patients with infratentorial location and those who received concomitant bevacizumab with radiation. Information about TTFields was provided at all facilities in accordance with the Japanese Society of Neuro-Oncology Guideline for appropriate use of TTFields. The final decision on whether to receive TTFields therapy was made by the patients themselves. We excluded patients who discontinued therapy prior to 3 months. Progressive disease was defined based on the response assessment criteria for neuro-oncology for high-grade glioma.^8^

The clinical data of patients included date of birth, sex, KPS before the start of adjuvant TMZ, preoperative and postoperative volume of contrast-enhanced lesions, extent of resection, histological diagnosis, genetic alteration, O6-methylguanine DNA methyltransferase (MGMT) methylation status, care giver, first date of TTFields usage, date of recurrence or progression, treatment after recurrence or progression, and date of death or last visit.

### TTFields Usage Rate

TTFields device accurately records the wearing time. Novocure Japan periodically downloads these records. The average percentage of daily use is reported monthly by Novocure Japan to prescribers and collected for all patients in this study. Compliance rates for TTFields use for the first 3 months were calculated as the average percentage of daily use. A compliance rate of TTFields ≥75% in the first 3 months is good tolerability and related to favorable prognosis in the final analysis of the EF-14 study.^3^ Therefore, we defined a compliance rate of TTFields ≥75% in the first 3 months as the threshold for high compliance here.

### Statistical Analysis

The PFS was calculated from the first date of craniotomy to the date of recurrence or progression. The OS was calculated from the first date of craniotomy to the date of death due to any cause or the last visit to our hospital. Nominal variables were analyzed using Fisher’s exact test, and continuous variables were analyzed using a *t*-test. Prognostic factors were analyzed using the log-rank test for the univariate analysis and Cox regression analysis for the multivariate analysis. Statistical significance was set at *P* < .05. Statistical analysis was performed using EZR statistical software (version 1.63).^9^

## Results

### Patient Characteristics

In total, 157 patients from 12 institutes received TTFields therapy. To examine the efficacy of TTFields, we excluded 41 (26%) patients who had discontinued during the first 3 months. The reasons for discontinuation were patients’ will (29 patients), progression of disease (11 patients), and wound infection (1 patient). Among the remaining 116 patients who used TTFields for at least the first 3 months, the median age was 57 years (range: 20-84 years), 67 (58%) were male, and the median KPS before the start of adjuvant TMZ was 90 (range: 40-100). The median follow-up duration was 18.2 months (range: 3.8-83.7 months). Gross-total removal, subtotal removal, partial removal, and biopsy were performed in 62 (53%), 23 (20%), 11 (9%), and 20 (17%) patients, respectively (Table 1).

**Table 1.** Characteristics of patients according to compliance rate of TTFields

Factors			Compliance rate of TTFields		*P* value
		*N*	≥75%	<75%	
Age	<65	77	47	30	.33
	≥65	39	20	19	
Sex	Male	69	39	30	.85
	Female	47	28	19	
KPS before the start of adjuvant TMZ	≤70	33	18	15	.68
	≥80	83	49	34	
Preoperative enhanced tumor volume	Average (cm^3^)		31.1	28.3	.65*
	Standard deviation (cm^3^)		34.4	30.6	
Postoperative enhanced tumor volume	0-1 cm^3^	72	45	27	.25
	>1 cm^3^	44	22	22	
Extent of resection	GTR	62	38	24	.82
	STR 80%-98%	23	13	10	
	PR 50%-80%	11	6	5	
	Biopsy or PR (<50%)	20	10	10	
Diagnosis by WHO 2021	Glioblastoma	99	57	42	.80
	IDH-mut Astrocytoma Gr. 4	10	6	4	
	Diffuse midline glioma	3	1	2	
	Diffuse hemispheric glioma	4	3	1	
MGMT methylation	Methylation	21	14	7	.66
	Unmethylation	27	16	11	
	Untested	68	37	31	
Care giver	Spouse	87	50	37	1.0
	Other than spouse	29	17	12	

Abbreviations: GTR, gross-total removal; IDH, isocitrate dehydrogenase; MGMT, O6-methylguanine DNA methyltransferase; OS, overall survival; PFS, progression-free survival; PR, partial removal; STR, subtotal removal; TTFields, tumor treating fields. *: t-test.

**Figure 1.**
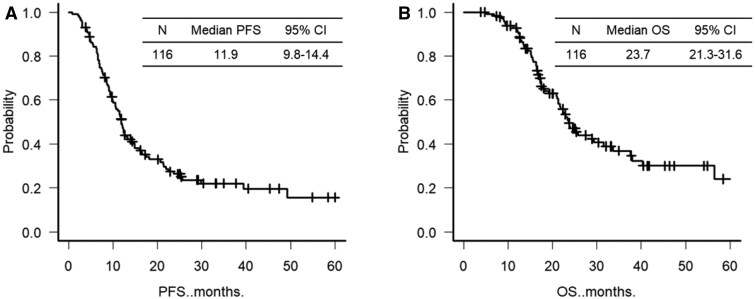
Kaplan–Meier survival curves for 116 patients who used TTFields for at least the first 3 months. (A) Kaplan–Meier curves related to PFS. (B) Kaplan–Meier curve related to OS. OS, overall survival; PFS, progression-free survival.

Regarding the integrated diagnosis based on WHO 2021 classification, glioblastoma, isocitrate dehydrogenase mutant astrocytoma grade 4, diffuse midline glioma H3-altered, and diffuse hemispheric glioma G34 mutant were present in 99 (85%), 10 (9%), 3 (3%), and 4 (3%) patients, respectively. The spouse assisted with the use of TTFields in 87 patients (75%). Median duration of TTFields use was 9.6 months (range: 3.0-68 months), and the average compliance rate was 78.3% (range: 5.7%-98%). Compliance rate of TTFields ≥75% for the first 3 months (high compliance) was achieved in 67 patients (67/116: 59%). Of the 94 patients who discontinued TTFields, 65 (69%) did so due to progressive diseases. In 116 patients who received TTFields for at least the first 3 months, the median PFS and OS were 11.9 months (95% CI, 9.8-14.4 months) and 23.7 months (95% CI, 21.3-31.6 months), respectively (Figure 1).

### PFS and OS

**Figure 2.**
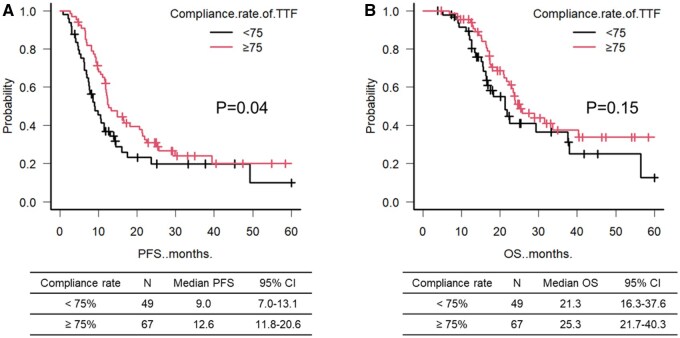
Kaplan–Meier survival curves according to compliance rate of TTFields. (A) Kaplan–Meier curve related to PFS. (B) Kaplan–Meier curve related to OS. CI, confidence interval; *N*, number; OS, overall survival; PFS, progression-free survival; TTFields, tumor treating field.

Univariate analysis of PFS was performed in 116 patients who used TTFields for at least the first 3 months. High compliance rate was the only significant prognostic factor (*P*  = .04) (Table 2 and Figure 2A). The median PFS for patients with high and low compliance rates was 12.6 and 9.0 months, respectively. Multivariate analysis was performed using 3 factors: KPS before the start of adjuvant TMZ, postoperative volume of contrast-enhanced lesion, and compliance rate of TTFields. Similarly, a high compliance rate was the only significant prognostic factor (Table 2). The baseline characteristics between patients with a high compliance rate and TTFields <75% (low compliance) were well balanced (Table 1). There were no significant differences between the 2 groups in age, sex, KPS before the start of adjuvant TMZ, enhanced tumor volume, extent of tumor resection, integrated diagnosis by WHO 2021 classification, degree of MGMT promoter methylation, and type of caregiver (all *P* > .05). In the univariate analysis of OS, a high compliance rate did not significantly prolong prognosis, although there was a tendency therein (*P* = .15) (Figure 2B). The median OS for patients with high and low compliance rates was 25.3 and 21.3 months, respectively. The median compliance rates during the first 3 months for patients with high compliance rates and low compliance rates were 87.7% and 58%, respectively. The median compliance rates during the whole period for patients with high compliance rates and low compliance rates were 78.4% and 50.1%, respectively.

**Table 2.** Univariate and multivariate analysis of progression survival in 116 patients who used TTFields for at least the first 3 months

		Univariate				Multivariate		
		*N*	Median PFS	95% CI	*P* value	Hazard ratio	95% CI	*P* value
Age	<65	77	11.8	9.8-14.9	.93			
	≥65	39	11.9	8.1-20.6				
Sex	Male	69	11.9	10.0-14.4	.47			
	Female	47	11.9	7.0-16.5				
KPS before the start of adjuvant TMZ	≤70	33	11.4	6.3-12.4	.09			
	≥80	83	12.2	9.8-16.1		0.69	0.43-1.10	.12
Postoperative enhanced tumor volume	0-1 cm^3^	72	12.6	10.6-17.4	.19	0.85	0.54-1.34	.48
	>1 cm^3^	44	10.5	7.4-12.4				
Diagnosis by WHO 2021	Glioblastoma	99	11.9	9.8-14.9	.66			
	IDH-mut Astrocytoma Gr. 4	10	17.4	4.0-NA				
	Diffuse midline glioma	3	10.7	5.4-NA				
	Diffuse hemispheric glioma	4	10.5	2.9-NA				
Care giver	Spouse	87	12.1	9.8-16.6	.12			
	Other than spouse	29	11.8	8.1-14.9				
Compliance rate of TTFields	≥75%	67	12.6	11.8-20.6	**.04**	0.64	0.41-0.99	**.04**
	<75%	49	9.0	7.0-13.1				

Abbreviations: CI, confidence interval; GTR, gross-total removal; IDH, isocitrate dehydrogenase; MGMT, O6-methylguanine DNA methyltransferase; OS, overall survival; PFS, progression-free survival; PR, partial removal; NA, not applicable; STR, subtotal removal; TTFields, tumor treating fields. Bold values indicate statistical significance set at *P* < .05.

### Treatment at First Recurrence or Progression

Among the 116 patients who used TTFields for at least the first 3 months, 86 patients (74%) experienced tumor recurrence or progression. Regarding treatment at first recurrence or progression, 47 (55%) patients received only chemotherapy with TMZ and/or bevacizumab, 18 (21%) received tumor removal, 12 (14%) received radiation therapy, and 9 (10%) received palliative care. Among patients with a high compliance rate, 49 patients relapsed: 22 (45%) received chemotherapy, 11 (22%) received tumor removal, 11 (22%) received radiation therapy, and 5 patients (10%) received palliative care. Among patients with a low compliance rate, 37 patients relapsed: 25 (68%) received chemotherapy, 7 (19%) received tumor removal, 1 (3%) received radiation therapy, and 4 (11%) received palliative care (Table 3).

**Table 3.** Treatment at recurrence or progression and proportion of patients

	Chemotherapy	Tumor resection	Radiation therapy	Palliative care
High compliance rate	45% (including BV: 86%)	22%	22%	10%
Low compliance rate	68% (including BV: 100%)	19%	3%	11%

## Discussion

In this retrospective study, 157 patients received TTFields therapy; 41 (26%) patients discontinued TTFields within the first 3 months. Additionally, only 43% of the patients (67/157) achieved a compliance rate of TTFields ≥75% for the first 3 months. Given that 75% of patients achieved a compliance rate of TTFields ≥75% for the first 3 months in the final analysis of EF-14,^3^ the compliance rate in our cohort was low. Several factors contribute to low compliance with TTFields therapy in Japan: the country’s hot and humid climate, discomfort, and skin irritation (eg dermatitis) caused by prolonged array wear, inconvenience of carrying the device during daily activities, and patients’ skepticism regarding the treatment’s effectiveness. To address these issues, recent developments have introduced softer and lighter high-flexibility electrode arrays, along with overall improvements in electrode weight and comfort. Moreover, it is essential for healthcare professionals and peer support networks to actively encourage patients by emphasizing the proven effectiveness and clinical necessity of TTFields therapy.

The EF-14 study showed that mild-to-moderate skin irritation from the TTFields transducer array occurred in more than half of cases.^3^ In addition to skin irritation, the continued application of the device on a shaved scalp can be burdensome for patients, leading to reduced adherence or discontinuation of treatment at the patient’s request. In our cohort, patient preference was the reason for 69% of those who discontinued TTFields therapy at an early stage of less than 3 months. To increase patient motivation for this therapy, providing numerical values for the duration and rate of use required for TTFields therapy to be effective is necessary.

PFS and OS in our cohort who used TTFields for at least 3 months were comparable to those in the EF-14 study.^3^ Median age, median KPS before the start of adjuvant TMZ, sex, and extent of tumor resection were also comparable to those in the EF-14 study. However, in actual clinical practice, it was found that a small number of patients with a KPS before the start of adjuvant TMZ of 60 or less and patients with WHO grade 4 gliomas other than glioblastoma received TTFields therapy. This is expected to be at the patient’s request, as treatment options for high-grade glioma are limited. In the future, the safety and efficacy of TTFields therapy in patients who are not included in the EF-14 inclusion criteria should be investigated.

Our study demonstrated that a compliance rate of  TTFields ≥75% in the first 3 months may be an independent factor to delay recurrence or progression. As shown in Table 1, there were no significant differences in characteristic factors such as age, KPS before the start of adjuvant TMZ, or residual tumor that may affect prognosis between patients with high compliance rates and those with low compliance rates. In some retrospective reports comparing TMZ maintenance therapy and TMZ maintenance therapy + TTFields therapy, the effects of TTFields therapy on PFS are inconsistent. A single-center report from China showed that TTFields therapy extended PFS (*P* = .041), especially in patients who underwent subtotal resection (*P* = .003).^6^ Conversely, a multicenter report from Japan showed that TTFields therapy did not extend PFS (*P* = .83).^7^ A major difference between these 2 reports was patient compliance with TTFields therapy. The average compliance rate in the Chinese cohort^6^ was high at 91.9%, but was 78.8% in the Japanese cohort.^7^ In this study, the median compliance rates for patients with high and low compliance rates were 87.7% and 58%, respectively. These results support that high compliance rate with TTFields therapy contributes to the extension of PFS.

Our study demonstrated that a high compliance rate tended to extend OS, but the difference was not significant. In our cohort, median survival postprogression was long at 12 months, which is similar to PFS. In a clinical study with a PFS benefit, a lack of statistical significance in OS did not demonstrate a lack of improvement in OS, especially for diseases with long survival postprogression.^10^ The number of cases in our study may be insufficient to demonstrate statistical significance.

As treatment for the first recurrence, radiation therapy was more frequently selected in patients with a high compliance rate than in patients with a low compliance rate (21% and 3%, respectively). As patients had already received extended local irradiation before TTFields therapy, re-irradiation for local recurrence carries a risk of symptomatic radiation necrosis.^11^ The use of TTFields therapy reduces local recurrence and increases distant recurrence.^12^ Furthermore, adding TTFields therapy to photodynamic therapy reduces distant recurrence in 80% of cases.^13^ In contrast, chemotherapy including bevacizumab was more frequently selected in patients with a low compliance rate than in patients with a high compliance rate (68% and 39%, respectively). This result is different from an interim analysis of the EF-14, showing 27% in the group with TTFields and 23% in the group without.^14^ Imaging data on tumor localization at the time of recurrence may further demonstrate the efficacy of TTFields as a localized treatment.

This multicenter retrospective study has some limitations. First, our cohort had insufficient information on MGMT methylation status, a significant prognostic factor for glioblastoma. Second, the sample size was relatively small. Third, due to the retrospective nature of this study, there is a possibility of selection bias.

## Conclusion

This multicenter retrospective study analyzed real-world clinical data on TTFields. A compliance rate of TTFields of 75% or higher during the first 3 months was significantly associated with improved PFS. A high compliance rate tended to extend OS, but the difference was not significant. A larger number of patients would allow for a more accurate assessment of the impact on survival of a TTFields compliance rate of 75% or higher during the first 3 months. Imaging data on tumor localization at the time of recurrence may further demonstrate the efficacy of TTFields as a localized treatment.

## Data Availability

The datasets generated and/or analyzed in the current study are available from the corresponding author upon reasonable request.
